# Interpersonal Influences on Body Representations in the Infant Brain

**DOI:** 10.3389/fpsyg.2018.02601

**Published:** 2018-12-21

**Authors:** Ashley R. Drew, Andrew N. Meltzoff, Peter J. Marshall

**Affiliations:** ^1^Institute for Learning and Brain Sciences, University of Washington, Seattle, WA, United States; ^2^Department of Psychology, Temple University, Philadelphia, PA, United States

**Keywords:** infants, EEG, somatosensory, touch, self, social perception, attention, interpersonal engagement

## Abstract

Within cognitive neuroscience, there is burgeoning interest in how the body is represented in the adult brain. However, there are large gaps in the understanding of neural body representations from a developmental perspective. Of particular interest are the interconnections between somatosensation and vision, specifically infants’ abilities to register correspondences between their own bodies and the bodies of others. Such registration may play an important role in social learning and in engendering feelings of connectedness with others. In the current study, we further explored the interpersonal aspects of neural body representations by examining whether responses to tactile stimulation in 7-month-old infants are influenced by viewing another’s body. During EEG recording, infants (*N*= 60) observed a live presentation of an experimenter’s hand or foot being touched. During the presentation of touch to the adult’s hand or foot, the infant received a brief tactile touch to their right hand or right foot. This resulted in four conditions: (i) receive hand stimulation/observe hand stimulation, (ii) receive hand stimulation/observe foot stimulation, (iii) receive foot stimulation/observe hand stimulation, and (iv) receive foot stimulation/observe foot stimulation. Analyses compared responses overlying hand and foot regions when the observed limb matched the stimulated limb (congruent) and did not match (incongruent). In line with prior work, tactile stimulation elicited a somatotopic pattern of results in the somatosensory evoked potential (SEP) and the sensorimotor mu rhythm (6–9 Hz). Cross-modal influences were observed in the beta rhythm (11–13 Hz) response and in the late potential of the SEP response (400–600 ms). Beta desynchronization was greater for congruent compared to incongruent conditions. Additionally, tactile stimulation to the foot elicited larger mean amplitudes for congruent compared to incongruent conditions. The opposite was true for stimulation to the hand. This set of novel findings suggests the importance of considering cross-modal effects in the study of neural body representations in the infant brain. Continued work in this new area of infant neuroscience research can inform how interpersonal aspects of body representations may serve to undergird early social learning.

## Introduction

The term “body representations” can refer to several different kinds of body-related constructs. One prominent approach to studying body representations has been to examine the neural mechanisms involved in the organization and maintenance of somatosensory processing in the brain. Most commonly, this pertains to the somatotopic representation of the body surface in primary somatosensory cortex, sometimes called a “body map.” A burgeoning aspect of this neuroscientific literature concerns the question of whether representations of one’s own body are connected with the representations of the body of others. In studies of human adults, it has been well documented that motor and sensory cortices allowing the control of movement and the registration of touch are also activated while observing others moving or being touched (Keysers et al., 2004; Rizzolatti and Craighero, 2004; Singer et al., 2004) and efforts have been made at modeling this (Pitti et al., 2013). This vicarious aspect of sensorimotor processing may draw on interconnections between vision and somatosensation, the study of which could provide insights into the origins and maintenance of interpersonal connectivity in early childhood (Marshall and Meltzoff, 2015; Meltzoff and Marshall, 2018).

Identifying self-other correspondences plays a role in social perception across the lifespan, and may be especially important for infants prior to language (Meltzoff, 2007). According to the “Like-Me” hypothesis (Meltzoff, 2007), the development of social cognition in infancy is grounded in the process of observing that others are similar to me at the level of bodily acts (Meltzoff, 2013). Such bodily connections between self and other may provide a foundation on which interpersonal relationships are built (Marshall and Meltzoff, 2014, 2015). One avenue for studying these aspects of body representations is the examination of how vision of others’ bodies influences the processing of tactile stimulation to one’s own body. The work presented here examines how brain responses to tactile stimulation of infants’ hands and feet are influenced by the vision of another person’s hand or foot being touched. At the highest level, the present work examines multimodal representation of the body in the infant brain.

In the current study we use the infant electroencephalogram (EEG) to investigate body representations. One advantage of employing EEG in the study of infant body representations is the temporally fine-grained way it allows for the examination of the processing of somatosensory stimulation. In turn, this temporal precision provides a window into different stages of somatosensory processing. In the current work, the influence of vision of other’s hands and feet being touched was tested for two aspects of infant EEG responses to tactile stimulation: (i) sensorimotor EEG oscillations, specifically the infant mu (6–9 Hz) and low beta (11–13 Hz) rhythms, and (ii) somatosensory evoked potentials (SEPs) elicited to touch. Both kinds of responses were examined at electrode sites overlying cortical sensorimotor regions, specifically central electrode sites. The three central electrode sites of interest in the current study were electrodes Cz (medial central) and C3 and C4 (left and right lateral central).

Previous work with infants has established that, in line with the somatotopic organization of somatosensory cortex found in adults, tactile stimulation of the right hand elicits the largest response in the infant EEG signal over the contralateral (left) electrode C3, stimulation of the left hand elicits a response over the contralateral (right) electrode C4, and tactile stimulation of the foot elicits a response at the midline central electrode (Cz) (Saby et al., 2015; Meltzoff et al., 2019). Further insights about somatotopy come from an EEG study of 6-7-month-old infants showing that the amplitude of the somatosensory mismatch negativity in infants is sensitive to the somatotopic arrangement of the body in primary somatosensory cortex (Shen et al., 2018).

The mu rhythm has frequently been employed to examine neural linkages between the execution of actions and the observation of similar actions (Fox et al., 2016). The infant mu rhythm occurs at a lower frequency range (6–9 Hz) than in adults (8–13 Hz) (Stroganova et al., 1999; Marshall et al., 2002). The beta rhythm (13–30 Hz in adults, lower in infants as noted below) also demonstrates consistent responses related to sensorimotor activity (for a review see Kilavik et al., 2013). In adults, beta power decreases during movement, tactile stimulation, and observation of actions, followed by a characteristic increase in beta power 300–1000 ms after stimuli completion that is known as the beta “rebound” (Gaetz and Cheyne, 2006; Caetano et al., 2007; Kilavik et al., 2013).

In the current study, we examined both mu and beta rhythms in response to tactile stimulation of infant body parts. While the boundaries of the infant beta rhythm have not yet been clearly established during infancy, visual inspection of our time-frequency plots showed time-locked activity in a low beta band (11–13 Hz). This aligns with expectations of rhythms occurring at lower frequency ranges during infancy compared to adulthood, although there is variability in approaches to delineating infant beta. Early and late windows of oscillatory responses were analyzed to account for power rebounds that are regularly observed in adults.

There is an established body of literature on EEG and MEG evoked responses to tactile stimulation in infants (Gondo et al., 2001; Nevalainen et al., 2008; Saby et al., 2015; Meltzoff et al., 2018, 2019). EEG studies reporting on the SEP response to tactile stimulation have found a large positivity occurring between 100 and 300 ms post-stimulus. For example, in a study of 7-month-old infants, Saby et al. (2015) observed a peak in the SEP at around 175 ms post-stimulus onset. In line with a somatotopic response pattern, the largest mean amplitudes of the early positivity to foot stimulation were found at electrode Cz, which overlies the foot region of sensorimotor cortex. Following hand stimulation, the largest responses were found over more lateral hand regions, with the response strongest at the site contralateral to tactile stimulation (C3 for right hand stimulation and C4 for left hand stimulation). A similar somatotopic pattern has been found in an EEG study of infants as young as 60 days of age (Meltzoff et al., 2019).

A series of recent studies has gone beyond unimodal tactile perception alone and provided evidence of a mapping between infants’ representations of their own body and the bodies of others by examining the effect of body-specific visual stimuli on sensorimotor EEG responses. In a live observation protocol, 14-month-old infants observed actions of an adult reaching toward and touching a toy using her hand or her foot (Saby et al., 2013). The infant mu rhythm response displayed a somatotopic pattern during the observation of the hand and foot actions, with greater mu desynchronization occurring over sensorimotor areas corresponding to the observed body part (i.e., a lateralized event-related desynchronization (ERD) response for hands and a medial response for feet). In a converging study using older infants, 12-month-old infants viewed videos of a human hand being touched or not touched (i.e., no contact was made) by an object (Müller et al., 2017). The extent of desynchronization of the infant mu rhythm over central-parietal sites was significantly greater when the human hand was touched. In a detailed MEG study of 7-month-old infants using source analysis, regions of cortex that were activated when the infant received a touch to the hand or foot were also found to be activated when watching a video of another person’s hand or foot being touched (Meltzoff et al., 2018). Taken together, these findings provide evidence for connections between the representation of the infant’s own body and the bodies of others.

We believe that a promising path toward enriching our understanding of infant body representations is to develop new paradigms for examining the multisensory integration of bodily information in young infants (e.g., Meltzoff and Marshall, 2018; Somogyi et al., 2018). Of particular interest are the temporal interactions between vision and somatosensation.

Adult studies have shown cross-modal effects such that viewing a body part modulates SEP responses to tactile stimulation while viewing the same part of one’s own body (Taylor-Clarke et al., 2002; Sambo et al., 2009; Cardini et al., 2012) and (to a lesser extent) while viewing the relevant part of another person’s body (Deschrijver et al., 2015; Adler et al., 2016). A similar body-specific visual modulation of neural responses to touch was demonstrated in a study of 3–4-year-old children using MEG (Remijn et al., 2014). To date, only one study has examined neural responses to simultaneous visual and tactile stimuli during infancy (Rigato et al., 2017). In this EEG study, 4-month-old infants viewed videos of a paintbrush touching an experimenter’s hand or the table surface next to the hand. The visual and tactile stimuli were synchronized such that infants received a vibrotactile pulse to the hand for 200 ms when the paintbrush made contact with the hand or the table. A positive peak in the SEP occurred within the first 200 ms after tactile stimulus onset, with significant differences in the amplitude of this peak occurring between the two conditions.

In the current study, we extended existing work by manipulating the correspondence of limbs in visual-tactile events in order to examine specificities of self-other body mappings in infancy. One novelty of the current study is that it used live visual presentations instead of video recordings, in order to attain greater ecological validity. Using a between-subjects design, 7-month-old infants received tactile stimulation to either their right hand or right foot. These touches occurred while infants observed an experimenter’s hand or foot being touched. This resulted in four conditions: (i) receive hand stimulation/observe hand stimulation, (ii) receive hand stimulation/observe foot stimulation, (iii) receive foot stimulation/observe hand stimulation, and (iv) receive foot stimulation/observe foot stimulation. We tested whether there were differences in the sensorimotor EEG rhythms (mu, beta) and SEP responses when the site of tactile stimulation to the infant was congruent with the site of observed stimulation, compared to when these sites were incongruent. Furthermore, we examined infants’ looking time to observing cross-modally congruent vs. incongruent displays.

## Materials and Methods

### Participants

Eighty-six infants were recruited from a diverse urban environment using commercially available mailing lists. All participating infants were born within 3 weeks of their due date and had not experienced serious developmental delays or illness. Infants taking long-term medication or who had two left-handed parents were excluded from the study. Twenty-six infants were not included in analyses due to insufficient trials remaining after rejection for movement artifact and/or lack of attention to the visual stimulus. The final participant sample comprised 60 infants (mean age = 6 months, 20 days; *SD* = 17 days). Within the final sample, 29 infants received stimulation to the right hand (19 females) and 31 infants received stimulation to the right foot (15 females).

### Tactile Stimulation

Tactile stimulation was delivered to the right hand or right foot of infants using an inflatable membrane mounted in a plastic casing (10 mm diameter; MEG International Services, Coquitlam, BC, Canada). A similar device for producing tactile stimulation has been used in prior EEG (Saby et al., 2015) and MEG studies (Pihko and Lauronen, 2004; Pihko et al., 2009; Meltzoff et al., 2018). Via flexible polyurethane tubing (3 m length, 3.2 mm outer diameter), the membrane was inflated by a short burst of compressed air controlled by STIM stimulus presentation software and a pneumatic stimulator unit (both from James Long Company, Caroga Lake, NY, United States).

For the delivery of tactile stimulation, a keypress by an experimenter triggered a solenoid to be opened on the pneumatic stimulator for 10 ms. This elicited an expansion of the membrane beginning 15 ms after trigger onset and peaking around 35 ms after trigger onset. The total duration of the membrane movement was around 100 ms. The 15 ms delay between trigger and membrane movement was corrected for in the timing of the events so that the time of 0 ms was the onset of membrane movement. The experimenter and pneumatic stimulator were located in an adjacent room behind a closed door to minimize audible solenoid operation in the testing room.

### Procedure

While seated on their caregiver’s lap, the infant’s head was measured and the infant was then fitted with an appropriately sized EEG cap. Tactile stimulators were attached at the midpoint of the dorsal surface of the right hand and right foot of the infant. The stimulators were attached using double-sided adhesive electrode collars in combination with medical tape, and then covered with a tubular bandage to hold them firmly in place, following the procedure used by Saby et al. (2015). A between-groups design was used to maximize the number of trials per condition. Infants were randomly assigned to one of two conditions: to receive stimulation to their hand or to receive stimulation to their foot. Infants sat on their caregiver’s lap throughout the experimental procedure. The caregiver was given instructions to prevent infants from putting objects in their mouth and to try to minimize extra movements.

#### Visual Stimuli

**FIGURE 1:**
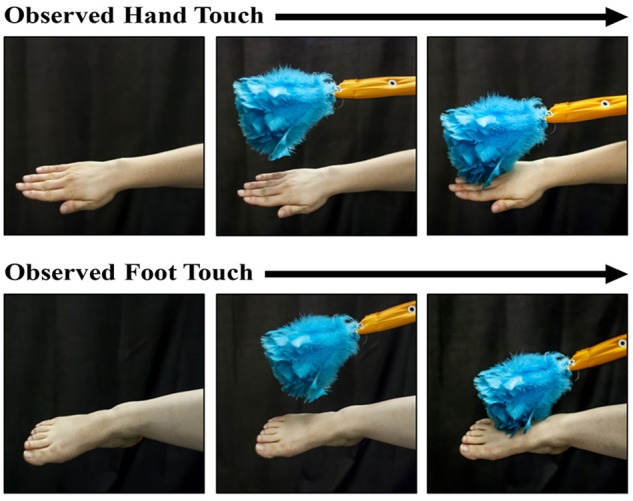
Photographs of the hand-touch and the foot-touch events observed by the infants during a trial. The presentations were accomplished by a live person and thus involved dynamic stimuli. A feather duster came into view and touched the experimenter’s hand or foot for the duration of two tactile stimulations to the infant’s right hand or foot (see text for stimulus-parameter details).

The protocol involved the coordinated work of three experimenters in order to achieve a well-controlled live 3-D display. Sitting behind a curtain, Experimenter 1 began by reaching beyond the curtain to display a spinning toy to attract the infant’s attention (∼56 cm away from the infant). Once the infant’s attention was obtained, Experimenter 1 retracted the toy and held out either her right hand or her right foot. Experimenter 2 (who was completely out of sight of the infant) accomplished a touch of the Experimenter 1’s hand or foot with a feather duster (see Figure 1) for approximately 3–4 s. While the feather duster was touching the hand or foot, Experimenter 3 (who sitting in an adjacent room and was observing a live video feed) twice triggered the opening of the solenoid, allowing the infant to receive two successive tactile stimulations (∼2 s apart). This process was repeated for a total of five times for a total of 10 tactile stimulations in one block. The blocks alternated between the display of the hand and foot of Experimenter 1 to the infants. The protocol contained a maximum total of 160 tactile stimuli (16 blocks), although the procedure was terminated if the infant could no longer maintain attention to the visual stimuli or became overly fussy.

#### Video-Recording of the Test Session

The experimental session was recorded on video for the purpose of coding infant attention and movement. A vertical interval time code (VITC) was placed on the video signal that was aligned with EEG collection at the level of one video frame. For each tactile stimulus, the epoch from -250 to 250 ms before and after the onset of the stimuli was coded offline for infant attention toward the experimenter’s hand or foot and large movements of the infant. Attention was coded if the infant maintained looking toward the hand or foot for the entirety of the epoch. Epochs were coded as containing large movements if they included gross body movements or large, repetitive movement of a limb (e.g., kicking a leg or batting a hand). Only trials in which the infant was attending to the visual stimulus were included in the final EEG analyses. In addition, trials containing large movements were excluded from the analyses. The video recording was also used to score the amount of time each infant spent looking at either the hand or foot of the experimenter during the experimental session.

### EEG Collection and Preprocessing

The EEG signal was recorded using a lycra stretch cap (Electro-Cap International) or a mesh stretch cap (ANT Neuro) with 21 electrodes (Fp1, Fp2, F3, F4, Fz, F7, F8, C3, C4, Cz, T7, T8, P3, P4, Pz, P7, P8, O1, O2, M1, M2) placed according to the 10–20 system. Scalp electrode impedances were accepted if they were at or below 35 kΩ. The signal from each electrode was amplified using optically isolated, custom bioamplifiers with high input impedance (>1 GΩ: SA Instrumentation) and digitized using a 16-bit A/D converter (±2.5 V input range). Bioamplifier gain was set at 4000 with hardware filter (12 dB/octave rolloff) settings at 0.1 Hz (high pass) and 100 Hz (low pass). During collection, the signal was referenced to the vertex (Cz) with an AFz ground.

The EEG Analysis System (James Long Company) and the EEGLab toolbox for MATLAB (Delorme and Makeig, 2004) were used for data processing. EEG signals were re-referenced to an average of the left and right mastoids. The signal was then low pass filtered at 30 Hz and segmented into 750 ms epochs for SEP computation and 2000 ms epochs for computation of event-related spectral perturbation (ERSP) in the mu (6–9 Hz) and low beta (11–13 Hz) bands. Epochs were visually inspected and excluded if they contained ocular or muscle artifact. Epochs were also excluded if amplitudes at central sites (C3, Cz, C4) exceeded ± 250 μV. Participants with less than nine trials within a condition after trial rejection were excluded from further analyses. After trial rejection, a 2(congruency) × 2(between-subjects limb stimulated) repeated-measures analysis of variance (ANOVA) was carried out on the number of trials included for the SEP and ERSP analyses. For each, there were significant main effects of congruency. There was a greater number of congruent trials compared to incongruent trials included in the SEP analyses [*F*_(1,58)_ = 13.50, *p* = 0.001; Congruent: *M* = 18.52; *SE* = 0.91; Incongruent: *M* = 15.92; *SE* = 0.89] and also for the ERSP analyses [*F*_(1,__52)_ = 6.19, *p* = 0.016; Congruent: *M* = 18.15; *SE* = 0.91; Incongruent: *M* = 16.36; *SE* = 0.96].

SEPs were computed for each participant relative to a prestimulus baseline of -100 to 0 ms, with time zero corresponding to the onset of membrane expansion at the skin surface. Participants with extreme SEP values (±40 μV) were not included in analyses. ERSP was calculated for the frequency range of 5–30 Hz using 100 overlapping windows starting with a 4-cycle wavelet at the lowest frequency relative to a prestimulus baseline of -500 to 0 ms. ERSP values for the mu (6–9 Hz) and beta (11–13 Hz) bands were then extracted. Extreme values (1.5× interquartile range) of the mu rhythm and beta rhythm ERSP responses for each condition, window, and electrode were not included in analyses.

### Statistical Analysis Plan

Analyses were time-locked to the onset of the tactile stimulation. During the window of analysis, the participants received the tactile stimulation while viewing the hand or foot of the experimenter being touched by a feather duster. The EEG analyses focused on a central region of interest (ROI) overlying sensorimotor regions, specifically electrodes Cz, C3, C4 (Saby et al., 2013, 2015). ERSP analyses examined an early (0–500 ms) and late (500–1000 ms) window of the mu and beta responses. SEP analyses examined the early positivity peaking between 100 and 300 ms and a late potential peaking within the window of 400–600 ms after the onset of tactile stimulation. Repeated-measures ANOVAs were carried out for each time window that included factors of limb-visual congruency (congruent/incongruent) x electrode (C3, Cz, C4) with a between-subjects factor of limb stimulated (hand/foot). The Greenhouse-Geisser correction factor was applied as appropriate. A repeated-measures ANOVA including the factors of limb-visual congruency and the between-subjects factor of limb stimulated (hand/foot) was also computed for infant looking time.

## Results

### Behavioral (Looking Time)

A repeated-measures ANOVA of infant looking time was conducted by calculating the percentage of time the infants were looking at the limb when it was visible (both congruent and incongruent limbs) as opposed to looking elsewhere about the room when a limb was visible. The ANOVA revealed no significant main effect of limb-visual congruency [*F*_(1,51)_ = 1.54, *p* = 0.22] or infant limb stimulated [*F*_(1,51)_ = 0.07, *p* = 0.79]. There was also no significant interaction [*F*_(1,51)_ = 0.07, *p* = 0.79].

### Mu Rhythm (6–9 Hz)

Tactile stimulation to the infant’s right hand and right foot elicited responses in the mu frequency band over the electrode sites of interest (C3, Cz, and C4).

#### Early Window (0–500 ms)

The repeated-measures ANOVA revealed a main effect of the limb stimulated on the infant [*F*_(1,52)_ = 8.78, *p* < 0.01]. Mu desynchronization was significantly greater for hand stimulation [*M* = -0.31; *SE* = 0.12] than foot stimulation [*M* = 0.18; *SE* = 0.12]. There were no other significant effects or interactions for the early time window.

#### Late Window (500–1000 ms)

The repeated-measures ANOVA for the late window revealed a main effect of electrode [*F*_(1.72,85.87)_ = 5.35, *p* = 0.01]. Pairwise comparisons revealed significantly greater mu desynchronization (*p* = 0.01) at C3 (*M* = -0.35; *SE* = 0.15) compared to C4 (*M* = 0.11; *SE* = 0.16). There was a main effect of the infant limb stimulated [*F*_(1,50)_ = 6.24, *p* = 0.02], with mu desynchronization being greater for hand stimulation (*M* = -0.46; *SE* = 0.18) than foot stimulation (*M* = 0.20; *SE* = 0.19). Finally, there was a significant interaction between electrode and the stimulated limb of the infant [*F*_(1.72,85.87)_ = 3.31, *p* = 0.05]. In the infant group receiving hand stimulation, greater desynchronization occurred at C3 (*M* = -0.87; *SE* = 0.21) than Cz (*M* = -0.46; *SE* = 0.21; *p* = 0.01) and C4 (*M* = -0.04; *SE* = 0.22; *p* = 0.001) and at Cz compared to C4 (*p* = 0.04). No other effects were significant.

### Beta Rhythm (11–13 Hz)

**FIGURE 2:**
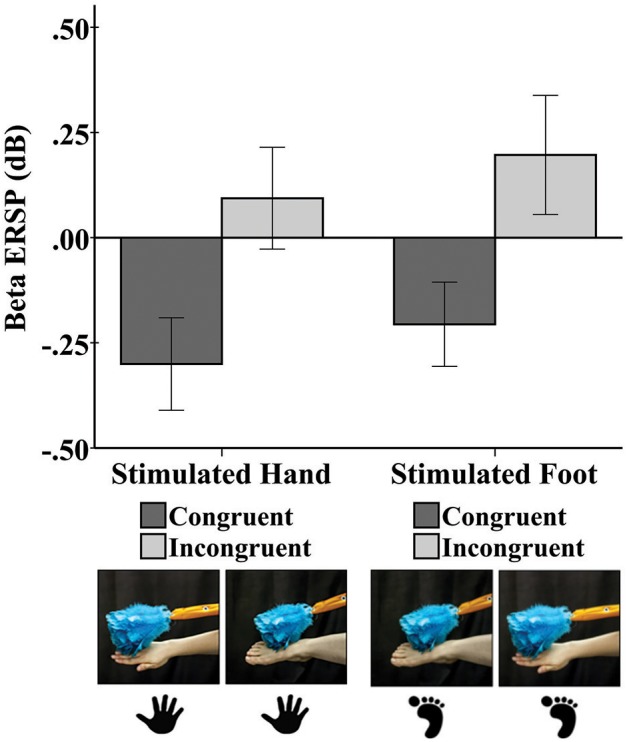
Mean beta rhythm ERSP at central electrode sites in response to tactile stimulation of the infant’s right hand and the infant’s right foot during the early time window (0–500 ms). Hand and foot icons indicate the infant body part receiving direct tactile stimulation. Photographs indicate which event the infant visually observed in the live demonstration. Error bars represent ± 1 standard error.

Tactile stimulation of infants’ right hands and right feet elicited responses in the beta frequency band at C3, Cz, and C4 (Figure 2).

#### Early Window (0–500 ms)

A repeated-measures ANOVA revealed a main effect of limb-visual congruency for the early window [*F*_(1,50)_ = 5.20, *p* = 0.03]. There was significantly greater beta desynchronization for the touch of the visual limb of the experimenter that was congruent with tactile stimulation on the infant’s own body (*M* = -0.23; *SE* = 0.11) compared to the touch of the visual limb that was incongruent (*M* = 0.15; *SE* = 0.14). Figure 2 shows the mean beta ERSP responses for each of the four conditions. No other effects were significant.

#### Late Window (500–1000 ms)

No effects or interactions were significant in a repeated-measures ANOVA examining the late window.

### Somatosensory Evoked Potentials

**FIGURE 3:**
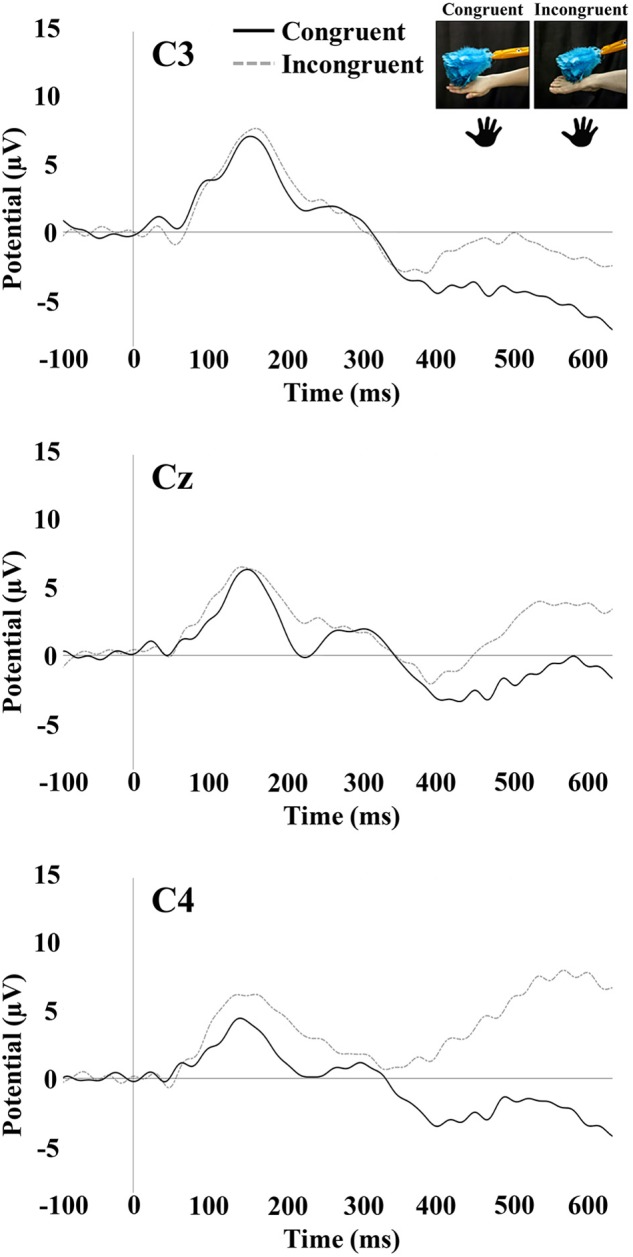
SEP responses to tactile stimulation of the infant’s right hand. The plots display responses at central electrode sites C3, Cz, and C4. Hand icons indicate the infant body part receiving tactile stimulation. The photographs depict the live visual event the infants observed.

**FIGURE 4:**
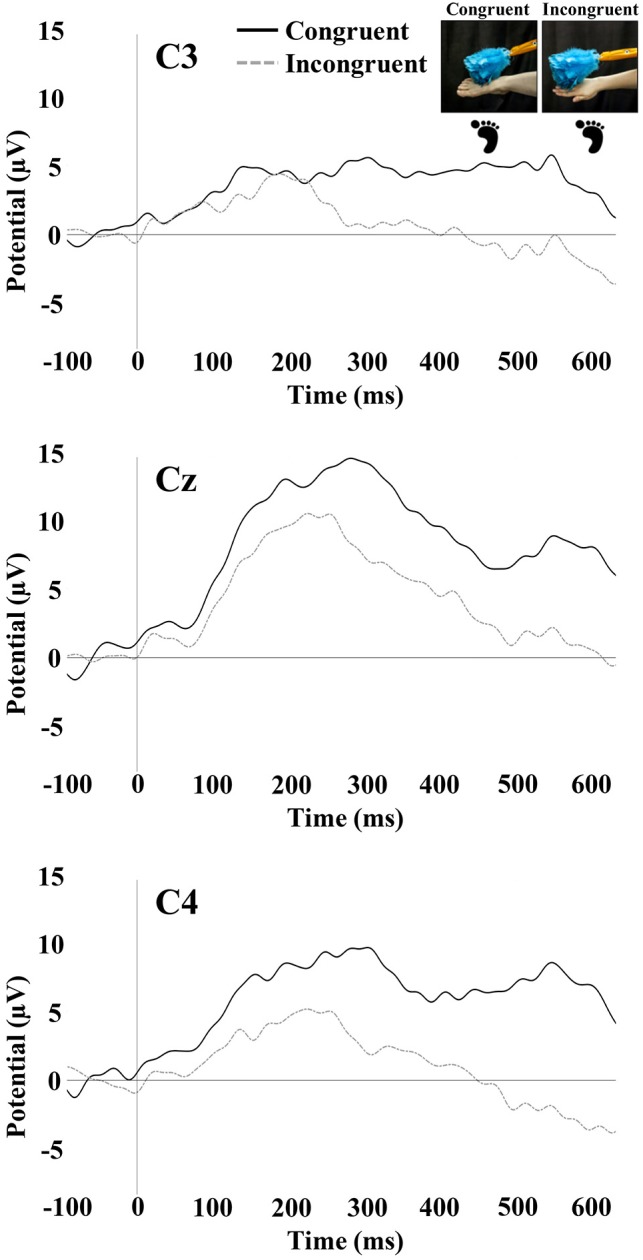
SEP responses to tactile stimulation of the infant’s right foot. The plots display responses at central electrode sites C3, Cz, and C4. Foot icons indicate the infant body part receiving tactile stimulation. The photographs depict the live visual event observed by the infants.

Tactile stimulation of the right hand and right foot of the infant elicited SEP responses that were examined at the central electrode sites C3, Cz, and C4. The SEP responses consisted of an early response at 100–300 ms and a later response at 400–600 ms. Figures3, 4 show the SEP responses at the three electrodes sites of interest for infant hand (Figure 3) and foot (Figure 4) stimulation.

#### Early SEP Positivity (100–300 ms)

The repeated-measures ANOVA for the early positivity revealed a significant main effect of electrode [*F*_(1.88,95.91)_ = 13.88, *p* < 0.001]. Pairwise comparisons revealed that mean amplitude at Cz (*M* = 6.78; *SE* = 0.68) was significantly greater than the mean amplitude at C3 (*M* = 3.97; *SE* = 0.85; *p* < 0.001) and C4 (*M* = 4.50; *SE* = 0.74; *p* < 0.001). There was a significant main effect of the stimulated limb of the infant [*F*_(1,51)_ = 6.54, *p* = 0.01]. The mean amplitude was greater for infant foot stimulation (*M* = 6.83; *SE* = 0.99) compared to infant hand stimulation (*M* = 3.34; *SE* = 0.94). There was also a significant interaction between electrode and stimulated limb of the infant [*F*_(1.88,95.91)_ = 16.98, *p* < 0.001]. Specifically in the infant group receiving foot stimulation, the mean amplitude at Cz (*M* = 10.25; *SE* = 0.99) was significantly greater than the mean amplitude at C3 (*M* = 4.16; *SE* = 1.23; *p* < 0.001) and C4 (*M* = 6.09; *SE* = 1.07; *p* < 0.001). In addition, the mean amplitude at C4 was significantly greater than the mean amplitude at C3 (*p* = 0.04). No other effects or interactions were significant.

#### Late SEP Potential (400–600 ms)

**FIGURE 5:**
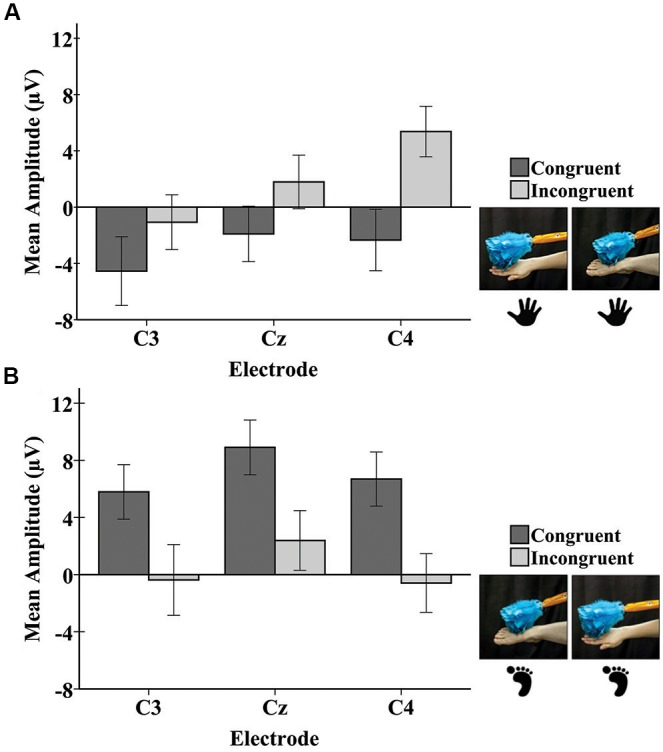
Mean amplitudes of the late potential in the SEP response at each electrode to tactile stimulation of the infant right hand **(A)** and the infant right foot **(B)**. Hand and foot icons indicate the infant body part receiving tactile stimulation. Photographs depict the live visual event observed by the infants. Error bars represent ± 1 standard error.

The repeated-measures ANOVA for the late potential revealed a main effect of electrode [*F*_(1.90,97.09)_ = 7.16, *p* < 0.01]. Pairwise comparisons revealed that the mean amplitude at C3 (*M* = -0.05; *SE* = 1.18) was significantly lower than at Cz (*M* = 2.80; *SE* = 1.09; *p* = 0.001) and C4 (*M* = 2.28; *SE* = 1.11; *p* = 0.01). There was a significant main effect of the stimulated limb of the infant [*F*_(1,51)_ = 4.31, *p* = 0.04], with the mean amplitude being greater for infant foot stimulation (*M* = 3.80; *SE* = 1.49) compared to infant hand stimulation (*M* = -0.45; *SE* = 1.41). There was a significant interaction between the limb-visual congruency and the stimulated limb of the infant [*F*_(1,51)_ = 11.31, *p* = 0.001]. For the infant group receiving foot stimulation, pairwise comparisons revealed a significant difference (*p* = 0.01) between the mean amplitudes of the congruent (*M* = 7.13; *SE* = 1.99) and incongruent (*M* = 0.48; *SE* = 1.90) conditions. This means that there was a greater mean amplitude for the touch of the visual limb of the experimenter that was congruent with the tactile stimulation on the infant’s own body (received foot/observed foot). For the infant group receiving hand stimulation, there was a significant difference (*p* = 0.04) between congruent (*M* = -2.93; *SE* = 1.88) and incongruent conditions (*M* = 2.03; *SE* = 1.79). In this case, the mean amplitude was greater for the touch of the visual limb of the experimenter that was incongruent with the tactile stimulation on the infant’s own body (received hand/observed foot). There was also a significant interaction between electrode and stimulated limb of the infant [*F*_(1.90,97.09)_ = 4.316, *p* = 0.02]. Specifically, in the infant group receiving hand stimulation, the mean amplitude at C3 (*M* = -2.81; *SE* = 1.62) was significantly lower than at Cz (*M* = -0.06; *SE* = 1.49; *p* = 0.02) and C4 (*M* = 1.52; *SE* = 1.52; *p* = 0.001). In the infant group receiving foot stimulation, mean amplitude at Cz (*M* = 5.65; *SE* = 1.58) was significantly greater than at C3 (*M* = 2.71; *SE* = 1.71; *p* = 0.01) and C4 (*M* = 3.05; *SE* = 1.61; *p* = 0.02). No other effects were significant. Figure 5 shows bar graphs of the average mean amplitudes of the late potential for each condition.

## Discussion

The current study examined whether infant neural responses to tactile stimulation of a specific body part were modulated by vision of the corresponding effector of another person. The primary aim of this work was to shed light on the suggestion that the infant brain registers correspondences between infants’ own bodies and the bodies of others (e.g., Marshall and Meltzoff, 2015; Meltzoff et al., 2018). This neuroscience work is relevant to theories of infant behavioral development and social perception. For example, an early-developing neural ability to detect interpersonal bodily similarities (e.g., between your own hand and the hand of another) may undergird imitative learning from others and promote social engagement between infants and caregivers by engendering feelings of connectedness.

The theorizing of Meltzoff (2007, 2013) posits that infants’ realization that others are “Like-Me” is a building block of early social cognition. It is hypothesized that this preverbal “Like-Me” recognition is supported by the fact body parts can be mapped as similar between self and other (Meltzoff and Moore, 1997, p. 186, Figure 2). The current study was aimed at harnessing methods from developmental cognitive neuroscience to further examine the interpersonal nature of infant body representations, primarily at the level of mapping the similarity between the observed body parts of others and felt body parts of self. More specifically, we tested infant neural responses to simultaneous visual and tactile stimuli, and examined whether the patterning of these responses was indicative of an interpersonal aspect of early body representations.

Independent groups of infants received tactile stimulation to either their right hand or right foot. Both groups observed a live-action presentation of an adult’s hand and foot being touched with a feather duster. This resulted in four conditions varying in visual-tactile congruency: (i) receive hand stimulation/observe hand stimulation, (ii) receive hand stimulation/observe foot stimulation, (iii) receive foot stimulation/observe hand stimulation, and (iv) receive foot stimulation/observe foot stimulation. A novel aspect of this study is that the tactile events were modeled by real people in a well-controlled live presentation. A comprehensive set of neuroscientific measures was used to investigate the temporal interactions between vision and somatosensation. Three types of neural responses were recorded: (i) the mu rhythm, (ii) the beta rhythm, and (iii) SEP responses at central electrode sites (C3, Cz, and C4). Analyses of the mu (6–9 Hz) and low beta (11–13 Hz) rhythms were split between early (0–500 ms) and late (500–1000 ms) windows following the onset of the tactile stimulation. SEP analyses focused on an early positivity occurring between 100 and 300 ms post-stimulus and a late potential between 400 and 600 ms. The discussion below first reflects on the somatosensory rhythm findings (mu and beta), then the SEP responses.

The mu rhythm response did not show an effect of congruency between stimulated and observed body parts for either the early vs. late windows. The main significant finding concerning mu was that in the late window, the mu rhythm showed a somatotopic pattern in which there was greater desynchronization at the contralateral electrode C3 for stimulation to the right hand and at the medial electrode Cz (compared to C4) for stimulation to the foot. Few studies have reported on the post-stimulus response of the mu rhythm following delivery or observation of a tactile stimulus. In adults, there is generally an initial decrease (ERD) in mu power that is characterized by a somatotopic scalp distribution. Mu rhythm desynchronization contralateral to the stimulated hand has been reported in MEG studies following punctate tactile stimulation in adults (Cheyne et al., 2003; Gaetz and Cheyne, 2006), sustained tactile stimulation (van Ede et al., 2014), and median nerve stimulation (Della Penna et al., 2004). The mu rhythm also shows contralateral desynchronization in anticipation of tactile stimulation to the hand, a finding that has been documented both in adults (Haegens et al., 2012) and children (Weiss et al., 2018). The current findings add a developmental perspective from infancy to work on mu rhythm responses elicited to tactile stimulation, and are also consistent with previously reported somatotopic mu rhythm patterns in older infants (12- and 14-month-olds). In these prior studies, the infant mu rhythm showed a somatotopic response during observation of another’s hand being touched (Müller et al., 2017), or another person reaching toward and touching a toy with their hand or foot (Saby et al., 2013).

We did not observe a somatotopic response pattern of the beta rhythm to tactile stimulation. However, there was an overall effect of limb-visual congruency in the early window of the beta rhythm response. This finding of differential modulation of the mu and beta bands may be related to reports in adults that both felt and observed touch activates a network of beta rhythm activity, but mu rhythm activity is more specific to felt touch (Pisoni et al., 2018). In the current study, there was greater beta desynchronization across the central region when infants were seeing a body part congruent with their body part being touched compared to seeing a different body part. This effect did not continue into the late window. The beta desynchronization elicited by the congruent condition resembles the desynchronization observed in adult studies following motor movement, action observation, and tactile stimulation (Cheyne et al., 2003; Gaetz and Cheyne, 2006; Kilavik et al., 2013). In the current study, the modulation of the early beta response by the congruency of the visual and tactile stimuli is notable. However, this modulation did not specifically vary by electrode.

The early positive peak of the SEP elicited in the current study is similar to the peak observed in a previous study of 7-month-old infants, which also showed a somatotopic response to hand and foot stimulation (Saby et al., 2015). In the current study, there was some evidence of a somatotopic response pattern for this peak when infants were being stimulated on the foot. However, a somatotopic pattern was not observed during hand stimulation, inasmuch as differences in mean amplitude of the early positivity were not significant between central sites. The lack of an observable somatotopic pattern in response to hand stimulation may be due to aspects of the experimental protocol that reduced somatotopic SEP responses. For instance, one important difference between the current study and prior infant EEG work (e.g., Saby et al., 2015) was the occurrence of tactile stimulation on different limbs in the prior study. In the current case, only one hand (the right hand) was stimulated throughout the entire experiment for the infants receiving hand stimulation. In the prior study, the right and left hand were stimulated as well as the right and left foot for each infant. It is possible that somatotopic responses in the prior work were more readily elicited by the variation (contrast) in the location of tactile stimulation and that in the current procedures, neural adaptation to hand stimulation may have occurred over the course of the experiment.

Examining a later window of the SEP response in infants showed findings for a late potential occurring between 400 and 600 ms post-stimulus. Effects of congruency on mean amplitude were observed in the late potential, with the specific pattern of effects being dependent on the infant limb stimulated. For stimulation of the foot, more positive mean amplitudes were elicited for congruent trials (i.e., during observation of the experimenter’s foot) than for incongruent trials. When the infant was receiving stimulation to the hand, more positive mean amplitudes of the late SEP potential were elicited for incongruent trials (i.e., during observation of the experimenter’s foot). These results are discussed further below.

To date, only two prior studies investigating SEP responses in infancy have reported a response at 400 ms or later after the onset of a tactile stimulus. The response observed in the studies by Rigato et al. (2014, 2017) showed positive peaks clearly between 400 and 600 ms, matching the timing of the current study but having a different appearance (as more of a positive peak). The late peak observed in the prior studies may be due to the use of long-duration, intense vibrating tactile stimulation lasting for 200 ms on the palms of the infants. Therefore, the late peak in the work of Rigato and colleagues may be a response to the termination of the tactile stimulation about 200 ms later.

The study of Rigato et al. (2017) also reported a difference between conditions when an infant observed a video of a hand being touched by a paintbrush vs. the paintbrush making contact with the table to the side of the hand. Unlike the current findings, this effect was observed in a much earlier window of the SEP, around 100 ms following tactile stimulus onset. Differences in the SEP between the current study and that of Rigato et al. (2017) could be due to a body-specific contrast (i.e., observing hand vs. foot, or observing hand vs. table) or, as previously mentioned, could be due to differences in tactile stimulation characteristics (vibrotactile stimulation). Similar to our current limb-visual congruency effect for infant hand stimulation, Rigato et al. (2017) found larger SEP responses at contralateral electrode sites when infants viewed the table being touched rather than the hand. In both studies, seeing another’s hand being touched while receiving tactile stimulation to the hand resulted in a suppression of the SEP response. Both studies therefore show that infant SEP responses can be affected by the observation of another’s body.

Differences found in the infant late potential related to the congruency between observed and stimulated body parts may be related to findings reported in adult work (Sambo et al., 2009; Longo et al., 2012; Deschrijver et al., 2015). In these adult studies, congruency effects were present in the SEP response after 200 ms post-stimulus bilaterally over central sites. It is conceivable that the late potential in infants (emerging at 400 ms) could be related to or even develop into the late positivity in the adult SEP response (emerging between 200 and 300 ms), reflecting a late stage of somatosensory processing.

The results of the current study suggest a discrepancy between infant foot stimulation and infant hand stimulation in the direction of the late potential modulation by limb congruency. While the late potential showed a larger positivity for congruent trials during foot stimulation, it was smaller for congruent trials during hand stimulation. One relevant factor could be the different SEP morphology observed in response to hand and foot stimulation (Figures 3, 4). The SEPs in response to foot stimulation show a very strong positivity particularly while infants were also viewing a foot, a pattern that persisted throughout the entirety of the SEP response. The SEPs in response to hand stimulation were slightly weaker and were less prominent across the overall time period analyzed. The reasons for these differences are uncertain, since they were not observed in Saby et al. (2015). One possible contributing factor is that the mean numbers of trials per condition were lower for the current study compared to the prior work. The protocol in Saby et al. (2015) had greater numbers of trials per limb because the tactile stimulation in that study was not systematically accompanied by congruent or incongruent visual input. Other possible explanations may be a novelty effect for tactile stimulation occurring on the dorsal area of the foot, or differential distortion effects occurring as the elicited electrical activity moves through the skull from the underlying sources. At a more psychological level, there are experiential differences between hands and feet for young infants. During the first year of life, infants are far more familiar with their own hands and viewing the hands of other people than they are with feet – infants regularly engage in own-hand regard, and the feet of others are more rarely viewed than their hands. The extent to which these and other developmental and experiential factors may contribute to the observed differences are topics for future research.

We also wish to draw attention to another aspect of the infant neuroscientific literature which is possibly relevant to the current work on infant neural body representations. Interestingly, studies examining ERP responses to visual stimuli in infancy have reported a component often referred to as the Nc (negative central) which occurs between 400 and 600 ms post-stimulus (Nelson and Salapatek, 1986; Richards, 2003; Reynolds and Richards, 2005; Wiebe et al., 2006; Ackles and Cook, 2007; Ackles, 2008). Although the morphology of the late potential observed in the current study does not necessarily resemble the large negative peak of the Nc, the onsets of the two potentials bear a resemblance to each other. Studies on the Nc have shown that it is modulated by factors such as frequency, and familiarity or novelty of the visual stimuli, such that the Nc is more negative for infrequent or novel stimuli. Attention toward the visual stimuli has also been shown to facilitate the Nc response in 4.5- and 7.5-month-old infants (Richards, 2003). Similarly, differences observed in the late potential of the current study may be due to body-specific attentional differences between infant’s viewing congruent and incongruent limbs.

Despite the importance of looking time measures in infant research more generally, neuroscience studies of infants have rarely included such measures. The current study included the scoring of infant looking time to the visual displays, as a complement to the electrophysiological measures. The results showed no significant difference between infant’s tendency to look at the experimenter’s limb when it was congruent or incongruent with the infant’s limb receiving tactile stimulation. Previous studies investigating body perception in younger infants found longer looking times toward congruent visual-tactile stimuli (Filippetti et al., 2013, 2015). In these studies, infants were touched on the face with a paintbrush while observing another face being touched. Infants looked longer when the observed face was being touched synchronously with the infant and in the same location (cheek or forehead). Therefore, very young infants may demonstrate increased visual attention to body-part correspondence between visual-tactile events under specific eliciting conditions (and perhaps using particular body parts, the face), which were not used in our current study.

The current neuroscience work can be connected, at least at a theoretical level, to three prominent lines of infant behavioral research, which also provide information about the role of the body in self-perception and interpersonal engagement. First, previous research has demonstrated infants’ ability to detect correspondences between their own seen and felt leg movements (Bahrick and Watson, 1985; Rochat and Morgan, 1995), which is compatible with the current findings of multimodal aspects of body perception. Second, research on infant facial and manual imitation suggests that infants can recognize correspondences between specific body parts of self and others (Meltzoff and Moore, 1997). In order to imitate with high fidelity, infants first need to identify which body part to use (tongue, fingers, lips) to generate the matching response, thus successful imitation provides a nonverbal indicator of interpersonal connectivity (Meltzoff and Marshall, 2018). Third, infant research also shows interpersonal coordination and adjustments to the body movements of others, for example, the findings that young infants make bodily adjustments in anticipation of a person approaching them in order to pick them up (Reddy et al., 2013). Taken together, these studies strongly suggest that infants’ coordination between their own body and those of others – which integrates tactile, proprioceptive, and visual domains in a multimodal fashion – is a fundamental and pervasive aspect of early development.

## Conclusion

The present findings contribute insights into how correspondences between vision and somatosensation may be processed by preverbal infants. This is a complex area that will benefit from detailed investigations of how different stimulus parameters influence infants’ neural responses. Based on the research reported here, some key factors that should be systematically manipulated in future neuroscience studies include: whether live or videotaped displays are shown, whether vibrotactile or punctate tactile stimulation of infants is used to provide tactile stimulation of the infant’s own body, whether effects differ by age and functional experience (e.g., differential experience between hands and feet), and whether one type of stimulus is repeatedly presented or infants have an opportunity to experience variation and contrasts between the stimuli.

We favor the idea that the body, even in infancy, is a multimodal rather than unimodal, construct (Meltzoff and Marshall, 2018). Young infants not only experience their own bodies but observe other people’s bodies and recognize similarities and differences between them. The neural representation of the body in the infant’s brain is a topic that addresses important issues in human development and promises to illuminate key aspects of social perception prior to language. Future work in this area will contribute to grounding the field of developmental social neuroscience, an area of research whose time has come.

## Ethics Statement

All subjects gave written informed consent in accordance with the Declaration of Helsinki. The protocol was approved by the Temple University IRB.

## Author Contributions

AD, AM, and PM were all involved in the conceptualization, design, analysis, and writing of the original research in the submitted manuscript. AD was involved in the collection of the data.

## Conflict of Interest Statement

The authors declare that the research was conducted in the absence of any commercial or financial relationships that could be construed as a potential conflict of interest.
